# P016. Congenital ataxia, hemiplegic migraine due to a novel mutation of CACNA1A: a case report

**DOI:** 10.1186/1129-2377-16-S1-A146

**Published:** 2015-09-28

**Authors:** Roberto Frusciante, Alessandro Capuano, Lorena Travaglini, Ginevra Zanni, Federico Vigevano, Enrico Bertini, Massimiliano Valeriani

**Affiliations:** Headache Center, Neurology Unit, Bambino Gesù Children's Hospital IRCCS, Rome, Italy; Unit of Molecular Medicine for Neuromuscular and Neurodegenerative Disorders, Bambino Gesù Children's Hospital IRCCS, Rome, Italy; Center for Sensory-Motor Interaction, Aalborg University, Aalborg, Denmark

## Background

The *CACNA1A* gene encodes the pore forming alpha-1A subunit of neuronal voltage-dependent P/Q-type Ca (2+) channels. Mutations in this gene result in clinical heterogeneity, including hemiplegic migraine, episodic ataxia, or progressive chronic conditions.

## Case report

An 8-year-old boy was admitted to our neurological unit due to an acute onset of left hemiparesis developed after a febrile episode. He also complained of headache with migraine characteristics. Brain MRI showed right hemispheric oedema. The hemiparesis disappeared completely after 1 week, and after steroid treatment. The patient was already known to our clinic since he was 2 years old when he was referred to us for a motor and cognitive developmental delay and for a cerebellar syndrome diagnosed as congenital ataxia. In the past all metabolic, biochemical and genetical analyses resulted negative. Serial brain MRI showed a progressive cerebellar atrophy. A *CACNA1A* gene mutation was hypothesised and sequence analysis revealed a heterozygous mutation c.4013C>T (p.I1338T) affecting the S4 segment and potentially damaging to the protein. This was a *de novo* mutation because it was not found in either parent.

## Conclusions

To the best of our knowledge this mutation of the *CACNA1A* gene has not been reported in the literature. Similar cases of a relatively long history of cerebellar ataxia, cognitive impairment and paroxysmal episodes are reported in the literature due to *CACNA1A* mutations. *CACNA1A* mutations present with a wide clinical spectrum. Congenital ataxia, mental retardation, and hemiplegic episode can be the presenting signs of *CACNA1A* mutations.

Written informed consent to publish was obtained from the patient(s).

